# Epigenetic modifiers *DNMT3A* and *BCOR* are recurrently mutated in *CYLD* cutaneous syndrome

**DOI:** 10.1038/s41467-019-12746-w

**Published:** 2019-10-17

**Authors:** Helen R. Davies, Kirsty Hodgson, Edward Schwalbe, Jonathan Coxhead, Naomi Sinclair, Xueqing Zou, Simon Cockell, Akhtar Husain, Serena Nik-Zainal, Neil Rajan

**Affiliations:** 1 Wellcome Trust Sanger Institute, Hinxton, UK; 2 Academic Department of Medical Genetics, University of Cambridge, Cambridge, UK; 3 MRC Cancer Unit, University of Cambridge, Cambridge, UK; 4 Institute of Genetic Medicine, Newcastle University, Newcastle upon Tyne, UK; 5 Department of Applied Sciences, Northumbria University, Newcastle upon Tyne, UK; 6 Northern Institute for Cancer Research, Newcastle University, Newcastle upon Tyne, UK; 7 Department of Pathology, Royal Victoria Infirmary, Newcastle upon Tyne, UK; 8 Department of Dermatology, Royal Victoria Infirmary, Newcastle upon Tyne, UK

**Keywords:** Cancer genetics, Skin cancer

## Abstract

Patients with *CYLD* cutaneous syndrome (CCS; syn. Brooke-Spiegler syndrome) carry germline mutations in the tumor suppressor *CYLD* and develop multiple skin tumors with diverse histophenotypes. Here, we comprehensively profile the genomic landscape of 42 benign and malignant tumors across 13 individuals from four multigenerational families and discover recurrent mutations in epigenetic modifiers *DNMT3A* and *BCOR* in 29% of benign tumors. Multi-level and microdissected sampling strikingly reveal that many clones with different *DNMT3A* mutations exist in these benign tumors, suggesting that intra-tumor heterogeneity is common. Integrated genomic, methylation and transcriptomic profiling in selected tumors suggest that isoform-specific *DNMT3A2* mutations are associated with dysregulated methylation. Phylogenetic and mutational signature analyses confirm cylindroma pulmonary metastases from primary skin tumors. These findings contribute to existing paradigms of cutaneous tumorigenesis and metastasis.

## Abstract (web summary)

*CYLD* cutaneous syndrome (also known as Brooke-Spiegler syndrome) is characterised by germline mutations in the tumor suppressor *CYLD*. Here, the authors highlight recurrent mutations in *DNMT3A* and *BCOR*, indicating a role for epigenetic dysregulation in this rare genetic skin disease.

## Introduction

In human skin, benign tumors outnumber malignant tumors, yet genetic studies of these are limited^1^. Rare inherited skin tumor syndromes such as *CYLD* cutaneous syndrome (CCS) offer an opportunity to address this knowledge gap and novel molecular insights into cancer can be gained. They may reveal unexpected driver mutations^2^, highlight mechanisms that may be targetable with repurposed drugs developed for other cancers^3^, or refine models of tumor growth and patterning. CCS patients develop multiple skin tumors named cylindroma, spiradenoma, and trichoepithelioma^4,5^, a histophenotypic spectrum of hair follicle-related tumors consistent with the hypothesis that they arise in hair follicle stem cells^6,7^. These tumors occur both at sun-exposed and sun-protected sites. Infrequently, salivary gland tumors, pulmonary tumors^8^, malignant transformation^9^, and metastasis with lethal outcomes can occur.

*CYLD* encodes a ubiquitin hydrolase enzyme involved in deubiquitination of lysine 63^10,11^ and Met 1-linked ubiquitin chains^12,13^. In CCS families, germline mutations occur within the catalytic domains of *CYLD* and are frequently truncating^14^, predicting loss of function. Loss of the wild-type parental allele (loss of heterozygosity (LOH)) of *CYLD* is demonstrated in the majority of inherited cylindromas, consistent with its role as a recessive cancer gene^15^. Genetic analysis of sporadic spiradenomas, rare in the general population, has highlighted mutations in *ALPK1* and MYB overexpression^16,17^. Taken together with the recent findings of upregulated MYB in CCS tumors, this supports MYB as a key downstream mediator of cylindroma pathogenesis following loss of *CYLD*^18^. However, beyond these drivers, CCS tumors studied using array-based comparative genomic hybridization demonstrate a paucity of DNA aberrations, restricted to copy-neutral LOH of *CYLD*^15^, incongruent with the diverse histophenotypes seen within and across tumor samples.

Arguably, *CYLD* loss alone may be sufficient for tumorigenesis, via its role in negatively regulating oncogenic pathways; CYLD depletion using RNA interference first revealed its role in negatively regulating nuclear factor-κB (NF-κB) signalling^10,11,19^. Corroborating this, murine *CYLD*-knockout models develop skin papillomas following chemical carcinogenesis that demonstrate increased expression of NF-κB target genes such as cyclin D1 (*CCND1*) mediated by dysregulation of BCL3^20^. Furthermore, CYLD has been shown to negatively regulate various oncogenic signalling pathways that are also relevant in hair development in embryogenesis, including Wnt^21^, Notch, and TGF-β^6^.

In humans, recurrent loss of functional CYLD is reported in diverse cancers, including myeloma^22^, leukemia^23,24^, hepatocellular carcinoma^25^, neuroblastoma^26^, and pancreatic cancer^27^, consistent with its role as a tumor suppressor expressed ubiquitously in normal tissues. In CCS patients, increased Wnt signalling has been shown to be an oncogenic dependency in cylindroma and spiradenoma tumors^7^. Histologically organized cylindroma and histologically disorganized spiradenoma represent extremes of a spectrum of histophenotype of the same tumor. Transition from cylindroma to spiradenoma is associated with loss of expression of the negative Wnt signalling regulator Dickkopf 2 (*DKK2*)^7^. DNA methylation has been suggested as a mechanism to account for loss of *DKK2* in a subset of samples studied^7^; however, comprehensive genomic and methylomic profiling of CCS tumors has not been performed. The inability of *CYLD*-knockout mouse models to recapitulate the human phenotype of cylindroma tumors has further limited characterization of the genetic drivers in CCS^6^.

In this study, we use whole-genome sequencing (WGS) and whole-exome sequencing (WES) to delineate the mutational landscape of CCS. We demonstrate a relative paucity of mutations in benign CCS skin tumors, among which epigenetic modifiers DNA methyltransferase 3a (*DNMT3A*) and BCL6 co-repressor (*BCOR*) are recurrently mutated. Malignant tumors in CCS have distinct driver mutations to benign tumors, and we track the origin of pulmonary cylindromas to the skin using mutation signature analysis. These findings in CCS advance our understanding of cutaneous tumorigenesis, pulmonary metastases, and malignant transformation.

## Results

### Biallelic loss of CYLD drives CCS tumors

**Fig. 1:**
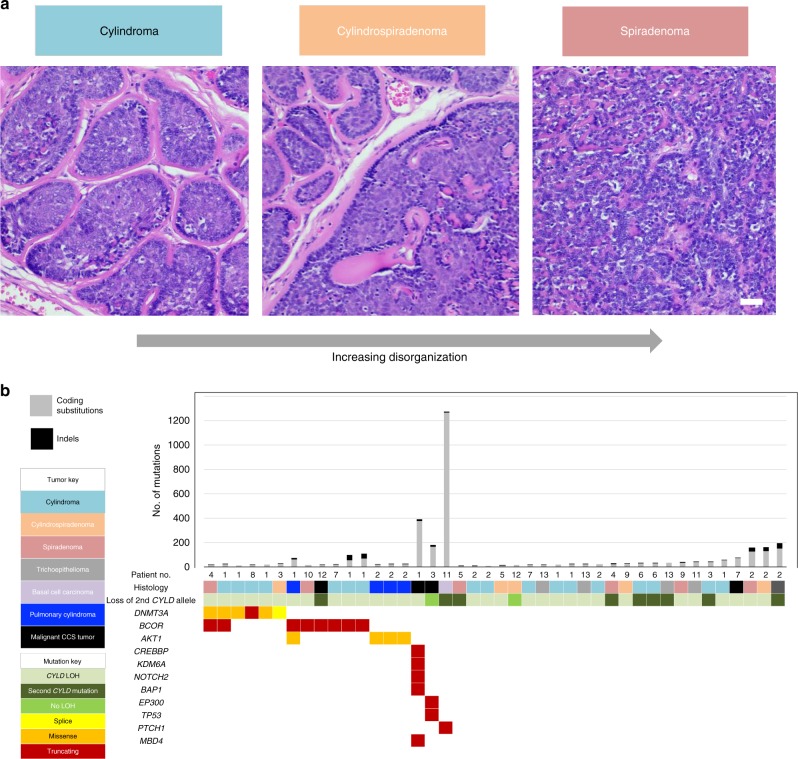
The mutational landscape of *CYLD* cutaneous syndrome. **a** Distinct histophenotypes of benign organized cylindroma and disorganized spiradenoma seen within the same sample, a frequent finding in CCS (white scale bar = 50 μm). **b** Epigenetic modifiers are mutated in CCS tumors. Mutational burden is indicated in the bar graph with corresponding mutated genes shown below in the matrix. Matrix rows indicate mutated genes in each tumor and each matrix column represents a different sample (*n* = 42)

To delineate the genomic landscape of CCS (Fig. 1a and Supplementary Fig. 1a–d) in humans, we studied DNA from 11 fresh frozen tumors using WGS in two directly related patients who had been under clinical follow-up for 35 years (patients 1 and 2) (Fig. 1b and Supplementary Data 1). The average number of unique reads per tumor and normal sample for WGS was 374,496,607, generating 35.5 mean fold coverage for all samples. We detected on average 1381 substitutions per tumor sample (average 0.44 mutations per Mb), 72 small insertions and deletions (indels), and 1 rearrangement, using WGS. Biallelic mutations in *CYLD* were a recurrent driver mutation, and no *MYB*-*NFIB* fusions were found, consistent with previous studies (Fig. 1b)^15^. Tumors demonstrated neither recurrent structural rearrangements nor recurrent copy number aberrations (Supplementary Fig. 2).

To validate these findings, we studied a further 31 tumors from 12 patients of 3 additional genotyped pedigrees using WES, given the lack of large structural rearrangements. We confirmed that *CYLD* biallelic loss was independent for each sample, reinforcing that each tumor arose independently: loss of the wild-type allele was observed either by LOH affecting 16q (31/42 tumors) or by a second mutation in *CYLD* (9/42), consistent with the loss of *CYLD* occurring across all benign and some malignant tumors in CCS.

### *DNMT3A* and *BCOR* are mutated in CCS tumors

**Fig. 2:**
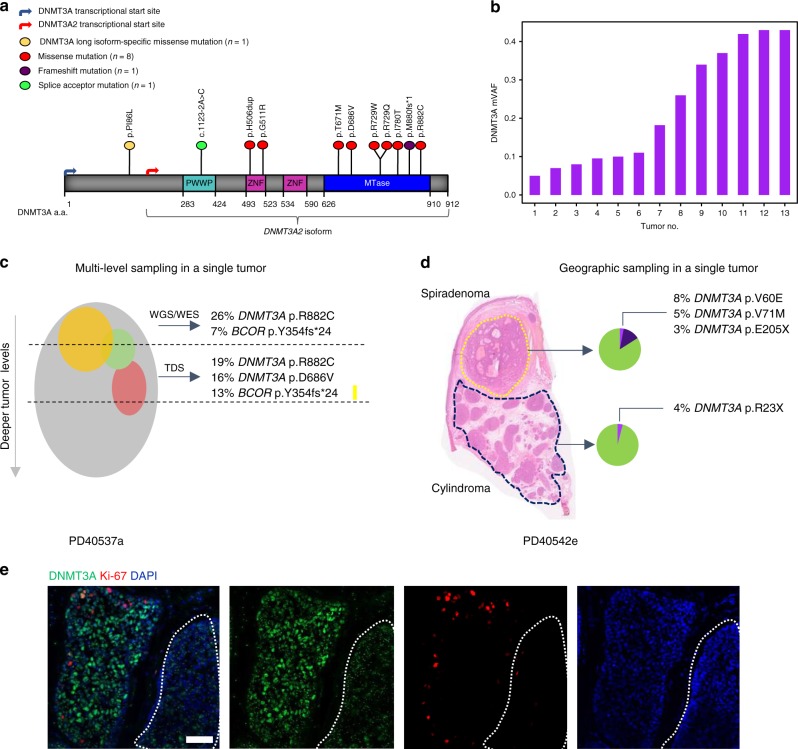
Intratumoral heterogeneity of *DNMT3A* mutation in CCS tumors. **a** *DNMT3A* somatic mutation lollipop diagram for CCS tumors. **b** Spectrum of mutant variant allele fractions (VAF) of tumors in this study. **c** Sampling of additional, deeper slices from a single tumor (PD40537a) reveals intratumoral heterogeneity of *DNMT3A* mutations (tumor indicated with gray sphere, intratumoral clones with colored spheres). **d** Geographic sampling of distinct histophenotypes (of cylindroma and spiradenoma) within a single tumor section (PD40542e) highlights marked clonal heterogeneity particularly of *DNMT3A* mutations. **e** Protein expression of DNMT3A and Ki-67 is variable within a “cylinder” of CCS tumor and across cylinders. An adjacent outlined cylinder of cells shows loss of DNMT3A expression (white scale bar = 50 μm)

In addition to biallelic mutations in *CYLD*, we discovered multiple mutations in epigenetic modifiers *DNMT3A* (*n* = 6) and *BCOR* (*n* = 8) in 12 tumors (Figs. 1b, 2a, Supplementary Fig. 3a, Supplementary Table 1, and Supplementary Data 2). In two tumors, both genes were mutated. *BCOR* mutations have been reported to co-occur with *DNMT3A* in over 40% of BCOR-mutated cases of AML, and a future larger study of these tumors may offer insights as to whether there is mutational synergy in CCS tumorigenesis^28^. Mutations in *DNMT3A* were predominantly missense mutations in the methyltransferase domain, but mutations in the zinc-finger domains were also noted and have been reported previously in COSMIC (Fig. 2a)^29^. Mutations in *BCOR* were predominantly frameshift mutations. Notably, different *DNMT3A* and *BCOR* mutations were seen in disparate tumors in patients 1 and 4, suggesting that convergent evolution drives tumorigenesis through epigenetic mechanisms in this cutaneous syndrome.

Interestingly, variant allele frequencies of *DNMT3A* and *BCOR* mutations ranged from 0.05 to 0.42—Fig. 2b), suggesting that intratumoral clonal heterogeneity may occur in these tumors. To explore this possibility, targeted deep sequencing (TDS; average coverage of >500×) of *DNMT3A* and *BCOR* was performed on additional material taken from further tissue sections of nine tumors studied above. This confirmed the presence of intratumoral heterogeneity of these putative driver mutations, with two distinct mutant clones or more found to co-occur within the same tumor in six samples (PD37330a, c, g, i, PD40536d, and PD40537a) (Fig. 2c and Supplementary Data 1).

To investigate whether *DNMT3A* mutational heterogeneity correlated with CCS tumor histophenotypes, we studied five tumors that contained intratumoral cylindroma and spiradenoma (Fig. 2d and Supplementary Fig. 4a, b). DNA was extracted from microdissected cylindroma and spiradenoma regions and TDS was performed. In three tumors, there was an identical *DNMT3A* mutation in both regions. In two tumors, there was heterogeneity between the histophenotypes, with private mutations in each regions, suggesting that multiple *DNMT3A* mutant clones of different sizes exist within tumors.

### Mutated DNMT3A2 dysregulates methylation

**Fig. 3:**
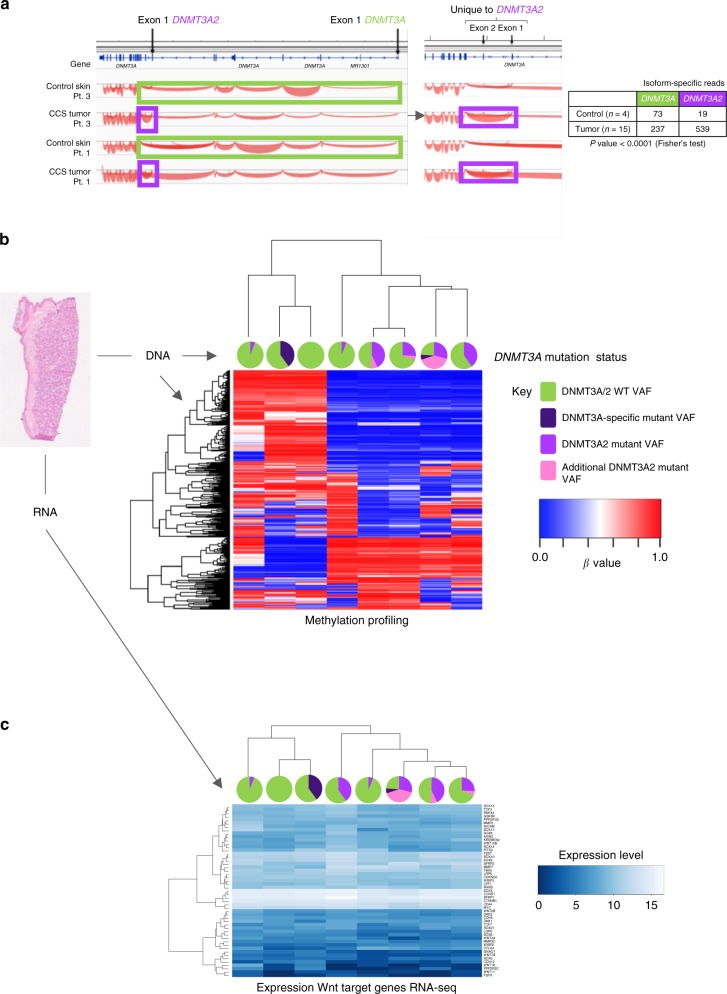
*DNMT*3*A*2 is overexpressed in CCS tumors. **a** RNA-sequencing of 15 CCS tumors revealed that the short isoform of *DNMT3A*, *DNMT3A2*, is preferentially overexpressed in CCS tumors. **b** In a further eight CCS tumors, DNA and RNA were extracted from the same sections. Methylation profiling, followed by unsupervised clustering of the 500 most variably methylated probes revealed two clusters (*DNMT3A* mutant VAFs are indicated as pie charts; heatmap key demonstrates β-values; blue indicates a low β-value (hypomethylated) and red indicates a high β-value (hypermethylation). **c** Expression of Wnt-β-catenin target genes in the same samples demonstrate the same two clusters are distinguished by expression levels of these genes

To explore the functional relevance of mutations in *DNMT3A* and *BCOR* in CCS tumors, RNA-sequencing was performed in 16 tumors. This revealed increased expression of the short isoform of *DNMT3A*, called *DNMT3A2*, in 15 tumors compared to four perilesional skin controls (Fig. 3a). DNMT3A protein expression was also increased in CCS tumors compared to control skin and hair, and regions of heterogeneity were observed between islands of cylindroma (Fig. 2e, Supplementary Fig. 5a, b). It should be noted that while this confirms protein expression, a caveat of these data is that the expression of DNMT3A may not reflect mutational and functional status. *BCOR* was expressed at similar levels in both control and tumor tissue (Supplementary Fig. 3b).

To assess the impact of *DNMT3A* mutations on methylation patterns, eight samples genotyped by TDS were studied using genome-wide DNA methylation arrays. Unsupervised clustering of the 500 most variably methylated loci revealed two clusters, one comprising five tumors with *DNMT3A2* isoform-specific mutations (*DNMT3A2*-mutated) (Fig. 3b). Comparison of these two clusters revealed 1512 differentially hypomethylated regions of contiguous probes in *DNMT3A2*-mutated tumors. Network analysis of these regions in *DNMT3A2*-mutated tumors identified the highest-ranked network to be functionally related to β-catenin (*p* < 1 × 10^−45^; Fisher’s exact test) (Supplementary Fig. 6 and Supplementary Data 3). Transcriptomic analysis of Wnt/β-catenin signalling pathway genes^30^ was performed on RNA extracted in parallel with DNA for the methylation analysis, as prior data in mouse skin showed *DNMT3A* loss is associated with dysregulation of multiple pathways including Wnt/β-catenin signalling pathway genes^30^ (Fig. 3c). This showed the same five tumors were distinguished as a cluster by Wnt/β-catenin target gene expression. This is an interesting preliminary finding in patient-derived tumors, and further functional studies will be needed to evaluate this association.

### Malignant CCS tumors carry epigenetic modifier mutations

Malignant transformation although uncommon in CCS is well-recognized. We studied five malignant CCS tumors: basal cell adenocarcinoma-low grade (BCAC-LG), malignant spiradenocarcinoma, atypical spiradenocarcinoma, poorly differentiated adenocarcinoma, and basal cell carcinoma (BCC) (Supplementary Fig. 7)^9^. The case of malignant spiradenocarcinoma (PD36119a) presented at the age of 80 in patient 1. The tumor had a comparatively high number of coding substitutions (375 in the exome, corresponding to 8.4 per Mb), consisting largely of C > T transitions at CpG dinucleotides. This hypermutator phenotype has been reported previously in conjunction with germline methyl-binding domain 4 (*MBD4*) mutations^31^. Closer inspection confirmed a germline *MBD4* mutation in the patient, with concomitant loss of the wild-type parental allele in the tumor. Cascade screening revealed other family members who also carried this variant (Supplementary Table 2), although their tumors did not have biallelic *MBD4* loss and thus did not have the associated mutational signature. The observed burden and pattern of mutagenesis was consistent with *MBD4*’s role as a DNA glycosylase safeguarding the integrity of methylated CpGs from deamination. Notably, additional mutations detected included epigenetic modifiers, *KDM6A* and *CREBBP*. Tumor suppressors *NOTCH2* and *BAP1* were also noted to be mutated.

Poorly differentiated adenocarcinoma (PD40536c) has not been reported in CCS and presented on the breast of a female CCS patient at age 47 years. The patient had extensive staging scans, mammograms, and biopsies of breast cylindromas, and has been followed up for 3 years with no evidence of a non-cutaneous primary tumor. This tumor had mutations in *TP53* and the epigenetic modifier *EP300*. Strikingly, this did not demonstrate LOH for *CYLD*. The BCAC-LG (PD40545a) tumor demonstrated a frameshift mutation in *BCOR*. The atypical spiradenocarcinoma (PD40540a) did not show any changes apart from *CYLD* LOH. The BCC (PD45044c) demonstrated a *PTCH* driver mutation and *CYLD* LOH, consistent with genetic features of BCC^32^. It also demonstrated the highest number of coding substitutions (1287) in our cohort, comprising the ultraviolet (UV) signature, in contrast to benign trichoepithelioma also arising on the face of the same patient. In summary, malignant tumors in CCS appear to have specific mutational patterns, and it would be interesting to determine if these tumor-specific mutations are recurrent in additional tumors in future studies.

### Pulmonary cylindromas originate from the skin

**Fig. 4:**
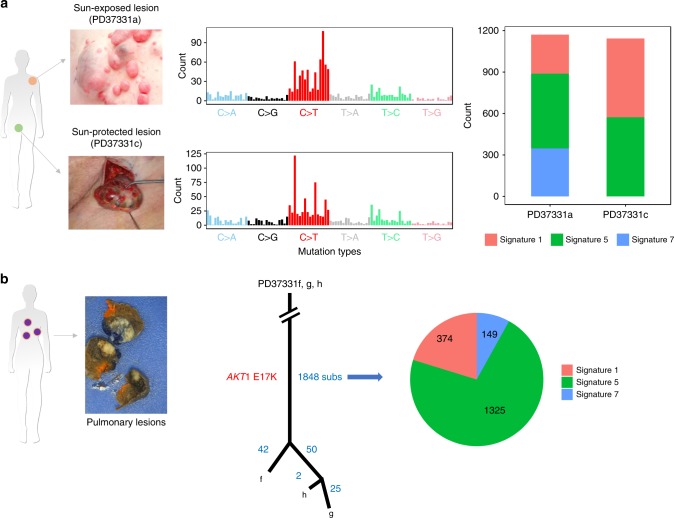
UV signature analysis reveals distinct mutational mechanisms in skin and tracks origin of lung tumors. **a** Examples of intermittently sun-exposed and sun-protected CCS tumors demonstrate differing mutational profiles. Mutational signature analysis reveals UV-related signature 7 in sun-exposed tumors only. **b** In one patient with three pulmonary lesions, a phylogenetic analysis reveals 1848 mutations were shared in common and showed a UV signature. Hence, these benign pulmonary lesions had a common origin, likely sun-exposed skin

To investigate mutational mechanisms that may give rise to the mutations detected in CCS patients, we compared the mutational signatures in tumors with identical histological types at intermittently sun-exposed and typically sun-protected sites^33^ (Fig. 4a). Two tumors from the torso demonstrated substitution signature 7 (*n* = 2; PD37331a, i) consistent with UV exposure. By contrast, we did not find evidence of signature 7 and found the presence of mutational signatures 1 (associated with deamination of methylated cytosines) and 5 (unknown etiology) in sun-protected tumors from pubic and perianal sites (*n* = 4; PD37330c, e, g and PD37331c) and some intermittently sun-exposed tumors from the breast and torso (*n* = 2). We surmise that in CCS, additional mechanisms other than UV are relevant to development of skin cancer.

We next used these data to investigate the concept of benign metastases seen in some patients with CCS, who develop multiple pulmonary cylindromas without typical features of malignancy^8^. We studied four pulmonary cylindromas that had benign histological features from patients 1 and 2, who were both ex-smokers. They did not have evidence of lymph node disease, hepatic, or bone metastases (Fig. 4b). Tumor phylogenetic analysis revealed that multiple pulmonary lesions from patient 2 shared 1848 substitutions, suggesting that these geographically separated lesions that seeded in the lung had a common origin. We found that the UV mutation signature 7 was present in the shared mutations, and thus tracked the origin of these pulmonary lesions to intermittently sun-exposed skin. Lastly, we found recurrent, E17K *AKT1* oncogenic mutations in multiple lung cylindromas in each patient, and in both patients independently as well. This is interesting for two reasons: First, although the numbers are small, this suggests that *AKT1* mutations likely arose prior to seeding in the lung. The *AKT1* mutations may confer lung tissue tropism for cylindromas. Second, this recurrent E17K *AKT1* mutation is clinically relevant and targetable. As drugs have been developed to target *AKT1* mutations in a diverse range of solid tumors^34^, this finding further creates therapeutic opportunities for this limiting secondary complication of CCS. It is of interest to note that three sporadic cutaneous spiradenomas also have recently been reported to carry this identical *AKT1* mutation (pulmonary status not reported) in the absence of a *CYLD* mutation^17^, suggesting that this finding may be relevant beyond CCS.

## Discussion

This work delineates the mutational landscape of CCS. A strength of our study is that we have employed WGS to comprehensively profile tumors from carefully phenotyped CCS patients, where long-term clinical follow-up date is available. Our work highlights the presence of distinct *DNMT3A* and *BCOR* mutations in different tumor sites of the same patient (inter-tumor heterogeneity) and different geographic sites within the same tumor (intra-tumor heterogeneity), which suggests strong convergent evolution (Supplementary Fig. 8) towards epigenetic dysregulation in this orphan disease where no medical treatments are available. In addition, we have performed matched analysis of methylome and transcriptome data in a subset of tumors, which offers insights in the absence of transgenic mice that recapitulate the human CCS phenotype. Finally, we uncategorically demonstrate that the multiple benign pulmonary lesions in this syndrome have a clonal, cutaneous ancestral origin—reinforcing the concept of benign metastases as a clinical phenotype.

Our data support a model where *DNMT3A2* isoform-specific mutations may selectively alter methylation in CCS tumors. We explored this in the context of Wnt/β-catenin pathway genes, as CCS tumor cells have a known Wnt dependency^7^; however, we could not conclusively prove a link between *DNMT3A* mutation and Wnt signalling using our models. It would be of interest to explore this potential association in mouse models in future studies, bearing in mind the caveat that existing *CYLD* mouse models fail to recapitulate the human phenotype of developing cylindromas. A separate limitation relating to the mutations detected in rare malignant CCS tumors is that future studies will be needed to demonstrate if the mutations found are recurrent.

Our findings may have clinical implications in the future. The *AKT1* mutation we report is targetable^34^, and is relevant to patients with pulmonary cylindromas carrying this change. Also, due to the clinical interest in mutated epigenetic modifiers in leukemia, strategies used to target *DNMT3A* mutant hematological malignancies may be relevant to CCS^35^. The accessibility of CCS skin tumors lend themselves to direct drug delivery, which may be an attractive route avoiding systemic side effects, as suggested by the methodology of a recent early phase clinical trial in CCS^3^.

## Materials and methods

### Patients and samples

Retrospective review of the case notes and radiological data of 15 genotyped CYLD mutation carriers that were under follow-up between 1 July 2013 and 1 July 2017 was performed. Skin and lung samples were obtained from patients with signed, informed, consent, and details of samples are shown in Supplementary Data 1. The authors affirm that human research participants provided informed consent for publication of the images in Fig. 4, Supplementary Fig. 1a, and Supplementary Fig. 7. Research ethics committee approval was obtained from the Hartlepool Research Ethics Committee and North East—Newcastle & North Tyneside 1 Research Ethics Committee for this work (REC Ref: 06/Q1001/59; 08/H0906/95 + 5).

### Histology and immunohistochemistry

Histological assessment was performed following standard hematoxylin and eosin (H + E) staining and in conjunction with a dermatopathologist (A.H.). Immunofluorescent labeling with antibodies against DNMT3A, β-catenin, and Ki-67 was performed^7^. Tissue sections from snap frozen skin tumor biopsies were fixed, blocked, and then probed overnight at 4 °C with primary antibodies. Antibodies against DNMT3A (#3598) and Ki-67 (#9449) were obtained from Cell Signalling, USA. β-Catenin antibody (#610153) was obtained from BD Transduction USA. Secondary fluorescent antibodies (Alexa Fluor #111-5451144 488-conjugated goat-anti-rabbit and #115-585-146 594-conjugated goat-anti-mouse) were applied the following day and visualized with a fluorescent microscope (Zeiss Axioimager Z2, with Apotome 2—Carl Zeiss, UK).

### Whole-genome sequencing and whole-exome sequencing

DNA was extracted from 12 cases along with corresponding normal tissue and subjected to paired-end WGS on an Illumina HiSeq X Ten^33,36^. DNA for WES was extracted from blood and cyrosections of snap frozen tissue, and in five cases from formalin-fixed paraffin-embedded tissue (PD37330h, PD40536c, PD40540a, PD40545a, and PD40545c). Forty-two WES library samples were prepared using the Illumina Nextera DNA Exome Kit, prior to being sequenced on a S2 flowcell on an Illumina Novaseq machine. Three WES samples were enriched using the SureSelect Human All ExonV6 + UTR and 100 base paired-end sequencing performed on an Illumina Hiseq 2500 genome analyzers. For WES sequence depth was on average 255**-**fold. Resulting BAM files were aligned to the reference human genome (GRCh37) using Burrows-Wheeler Aligner, BWA-0.7.16a (r1181). Mutation calling was performed using CaVEMan (Cancer Variants through Expectation Maximization: http://cancerit.github.io/CaVEMan/) for calling somatic substitutions^33^. Indels in the *tumor* and normal genomes were called using a modified Pindel version 2.0 (http://cancerit.github.io/cgpPindel/) on the NCBI37 genome build. Structural variants were discovered using a bespoke algorithm, BRASS (BReakpoint AnalySiS; https://github.com/cancerit/BRASS) through discordantly mapping paired-end reads followed by de novo local assembly using Velvet to determine exact coordinates and features of breakpoint junction sequence. All mutations were annotated according to ENSEMBL version 75.

### ASCAT copy number analysis

Allele-specific copy number analysis of tumors analyzed by WGS was performed using ASCAT (v2.1.1)^33^. ASCAT takes non-neoplastic cellular infiltration and overall tumor ploidy into consideration, to generate integer-based allele-specific copy number profiles for the tumor cells. Copy number values and estimates of aberrant tumor cell fraction provided by ASCAT were input into the CaVEMan substitution algorithm for WGS. In addition, ASCAT segmentation profiles were used to establish the presence of LOH across *CYLD* and relevant mutated cancer driver genes.

### Identification of driver mutations

Somatic mutations present in known cancer genes (Cancer gene census https://cancer.sanger.ac.uk/census) were reviewed to identify those which were likely to be driver mutations. Mutations were deemed to be potential driver mutations if they were consistent with the type of mutations found in a particular cancer gene, that is, inactivating mutations in tumor suppressor genes (including nonsense, frameshift, essential splice site mutations, and recurrent missense) and recurrent mutations in dominant oncogenes. Recurrent mutations were determined by reference to reported mutation frequency in the COSMIC database (https://cancer.sanger.ac.uk/cosmic).

### Mutational signature analysis

The contributions of substitution signatures for WGS samples were determined as follows: the substitution profile is described as a 96-channel vector. For each mutation, of which there are six substitution classes of C > A, C > G, C > T, T > A, T > C, and T > G, the flanking 5′ and 3′ sequence context is taken into account giving a total of 96 channels. A given set of mutational signatures was fitted into the mutational profile of each sample to estimate the exposure of each of the given signatures in that sample. The fitting algorithm detects the presence of mutational signatures with confidence, using a bootstrap approach to calculate the empirical probability of an exposure to be larger or equal to a given threshold (i.e., 5% of mutations of a sample). Here, we first used 30 COSMIC signatures (https://cancer.sanger.ac.uk/cosmic/signatures) to fit into each sample, and then chose the first three signatures with highest confidence, which are signature 1, 5, and 7, to do the final fitting.

For highly mutated malignant samples (the spiradenocarcinoma (PD36119a) and the BCC (PD40544c)), the mutation burden was orders of magnitude higher than other non-malignant tumors that were exome sequenced. We were able to use cosine similarity between the overall 96-channel profile and COSMIC signature to confirm the presence of particular mutational signatures in the relevant sample. The cosine similarity between each malignant sample and the suspected COSMIC signature was high: for PD36119a, cosine similarity to COSMIC signature 1 was 0.92 and for PD40544c cosine similarity to the UV light signature, COSMIC signature 7, was 0.98.

### Targeted sequencing

The Truseq Myeloid panel (Illumina) was used to sequence *DNMT3A* and *BCOR* in 18 samples in accordance with the manufacturer’s protocol. A 20 pM library of the PhiX genome was added to achieve a 5% PhiX spike-in. This library was loaded onto a Miseq flowcell (600 cycles V3) for sequencing (Illumina, San Diego, CA, USA). Data were analyzed using BWA (v.0.7.15) to align reads to the reference sequence and Samtools used as a variant caller. Variant calls that passed strict filtering thresholds (“Filter” = PASS and “Qual” = 100) were included for the deep sequencing on sections in additional levels and in new samples. For five samples (PD37330k, PD37331k, PD37331m, PD40542e, and PD 40536e—Supplementary Fig. 4) where intratumoral clonal variation was studied across distinct histophenotypic regions, variant call thresholds were relaxed, and all non-synonymous variants called were confirmed by visualizing aligned read data using Integrated Genomics Viewer (IGV; v2.3). These variants were included if aligned reads supported the variant calls.

### Transcriptomic analyses

RNA was extracted from 16 tumor samples and 4 control samples and stranded preparation was performed using the Illumina Stranded mRNA Kit^3^. Libraries were prepared and sequenced using an Illumina Hiseq 2500, giving 15 million paired-end reads per sample, which were 100 bp in length. For eight additional samples (PD37330a, c, e, k, PD40539d, e, and PD40542d, where DNA and RNA were extracted from the same cells), libraries were generated using the NEB Nextera Low Input RNA Library Prep Kit, and were sequenced using an Illumina Novaseq 6000. FASTQ files were aligned using the splice aware aligner program STAR to generate alignment files^37^. The read counts for each sample file were counted using the R package Subread^38^. Differential gene expression analysis was carried out using the package DeSeq2^39,40^. Log-transformed count matrix values were used for heatmap generation using the gplots^41^ package.

### Methylation assay and analysis

We assessed genome-wide DNA methylation in eight tumor samples with the Illumina Methylation EPIC microarray (Illumina, San Diego, CA, USA). DNA methylation assays were performed as per the standard manufacturer’s protocol by MWG (Aros, Denmark). Briefly, these are eight CCS tumors in which detailed analysis was performed as follows. DNA and RNA were extracted from the same cells, and mutation status of *DNMT3A* and methylation profiling were performed. Methylation array processing, functional normalization^42^, and quality control checks were implemented using the R package minfi^43^. Differentially methylated probes were identified using minfi. Differentially methylated regions spanning multiple probes were identified using bumphunter;^44^ these regions were visualized using Gviz^45^. When these methylation profiles were assessed, the 500 most variably methylated probes were subject to unsupervised hierarchical clustering. The study of the 500 most variable probes is an accepted approach to help distinguish methylation profiles of tumors^46^. A Euclidean distance matrix was constructed and hierarchical clustering was subsequently performed using the “complete” agglomeration method. The 500 probes with the highest standard deviation were selected for visualization. This analysis demonstrated that the majority of *DNMT3A2*-mutant tumors clustered separately from *DNMT3A2* wild-type tumors (Fig. 3b). We then studied these two groups and assessed all genes related to probes that were significantly differentially methylated between these two clusters with a *p* value of <0.05. Network analysis of these genes using Ingenuity Pathway Analysis^47^ revealed networks of genes related by function that were ranked by *p* value (Supplementary Data 3).

### Reporting summary

Further information on research design is available in the Nature Research Reporting Summary linked to this article.

## Supplementary information

Supplementary Information

Description of Additional Supplementary Files

Supplementary Data 1

Supplementary Data 2

Supplementary Data 3

Reporting Summary

## Data Availability

The WGS data have been deposited in the European Genome-phenome Archive (EGA) database under the accession code EGAD00001004573. The WES data have been deposited in the EGA database under the accession code EGAD00001005305. RNA-sequencing data have been deposited in the EGA database under the accession code EGAD00001005305. TDS data were deposited in the EGA database under the accession code EGAD00001005305. Methylation data have been deposited in the EGA database under the accession code EGAD00010001755. All the other data supporting the findings of this study are available within the article and its Supplementary Information files and from the corresponding author upon reasonable request.

## Code availability

No new code was produced for the analysis of the data in this manuscript. Details of the computer code used are included in the Methods section.
