# Supplementary material for: Epigenetic modifiers DNMT3A and BCOR are recurrently mutated in CYLD cutaneous syndrome
Source: Nat Commun. 2019 Oct 17;10:4717. doi: 10.1038/s41467-019-12746-w (PMC6797807; doi:10.1038/s41467-019-12746-w)
Supplement: Supplementary file 1 — Supplementary Information [file 41467_2019_12746_MOESM1_ESM.pdf]

Supplementary Information for Davies *et al.*

Epigenetic dysregulation underpins tumorigenesis in a cutaneous tumor syndrome.

*Corresponding authors: Neil Rajan, Institute of Genetic Medicine, Newcastle University, NE1 3BZ, UK. Tel : +44 191 2418813. Email: neil.rajan@ncl.ac.uk ORCID ID: orcid.org/0000-0002-5850-5680 and Serena Nik-Zainal, Academic Department of Medical Genetics, University of Cambridge, Cambridge, UK. Email: Serena Nik-Zainal snz@mrc-cu.cam.ac.uk

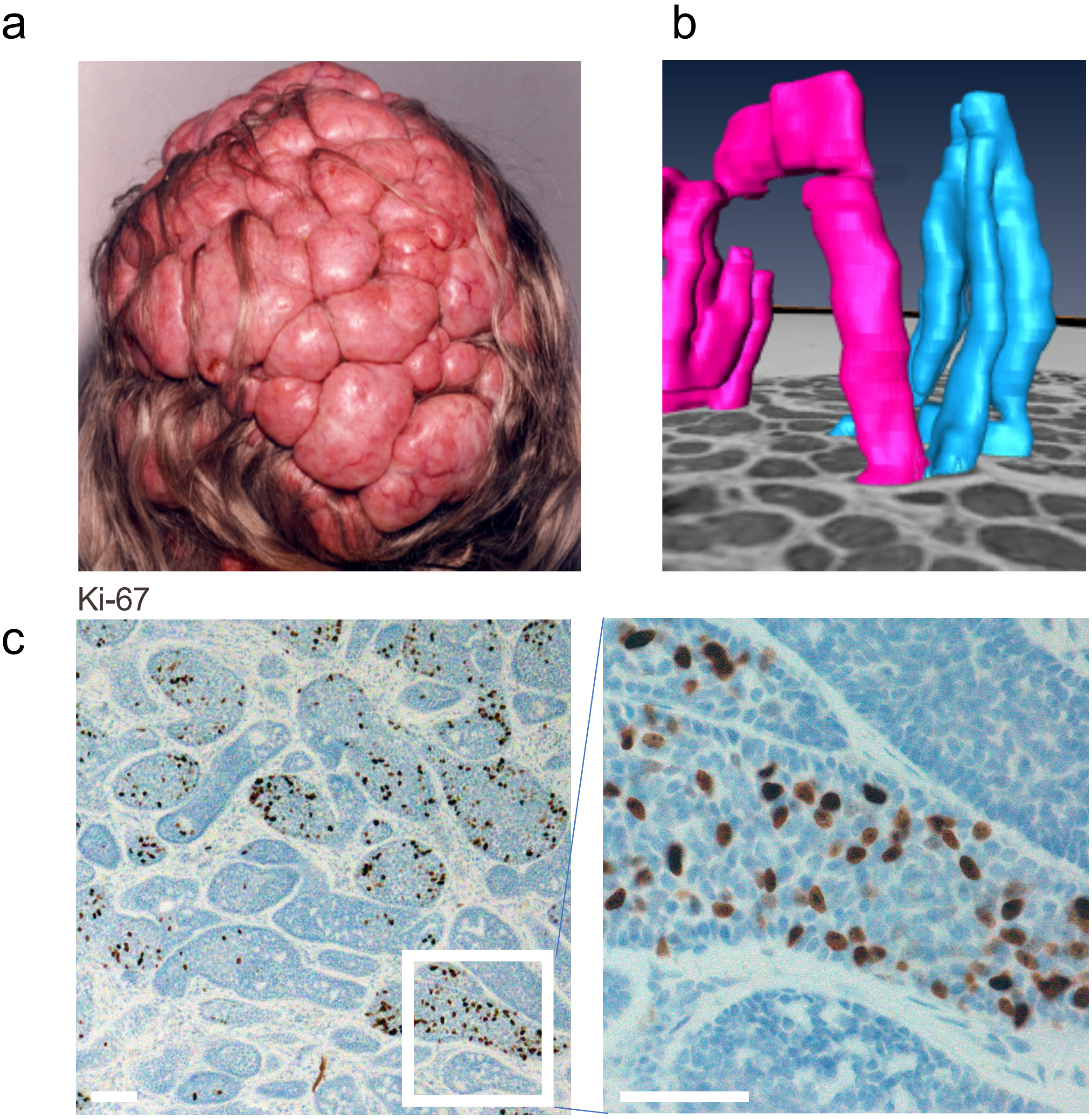

Supplementary Figure 1. Histological patterns of growth of CCS tumors. a, Benign but disfiguring multiple cylindroma skin tumors of the scalp. **b,** Three-dimensional reconstruction of tumors from serial sections *in silico* highlight adjacent islands of tumor as distinct cylindrical compartments indicated in blue and red, with (**c)** a range of states of proliferation as indicated by expression of the proliferation marker Ki-67 (White scale bar=100μm).

a

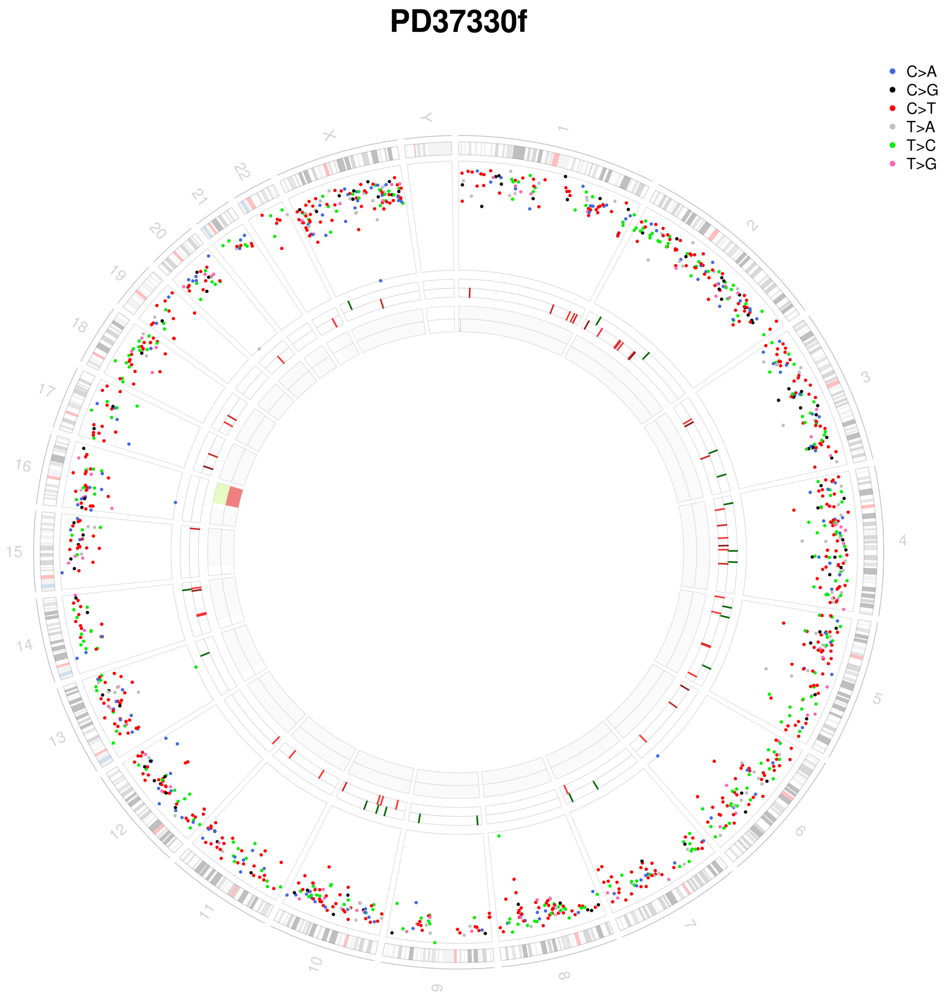

Supplementary Figure 2. Loss of heterozygosity affecting 16q is a recurrent feature in CCS tumors. a Whole genome circos plot depicting from outermost rings heading inwards: Karyotypic ideogram outermost. Base substitutions next, plotted as rainfall plots (log^{10} intermutation distance on radial axis, dot colours: blue, C>A; black, C>G; red, C>T; grey, T>A; green, T>C; pink, T>G). Ring with short green lines, insertions; ring with short red lines, deletions. Major copy number allele ring (green, gain), minor copy number allele ring (red, loss).

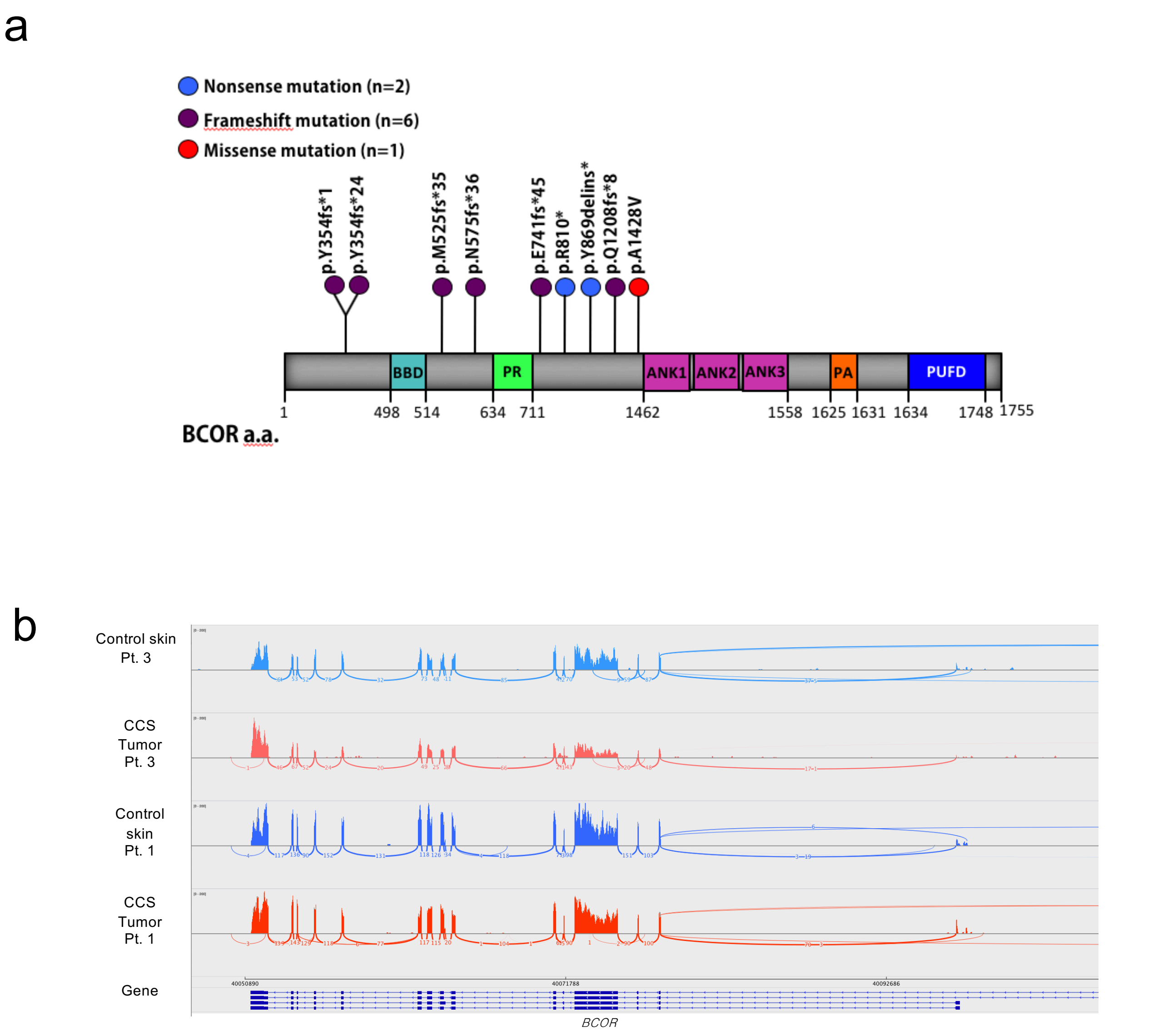

Supplementary Figure 3. Mutation spectrum of *BCOR* and expression in CCS. **a** Lollipop diagram indicating the distribution of mutations in *BCOR*. **b** RNA sequencing data demonstrating indicative expression of *BCOR* in 2 CCS tumors and matched patient controls.

a

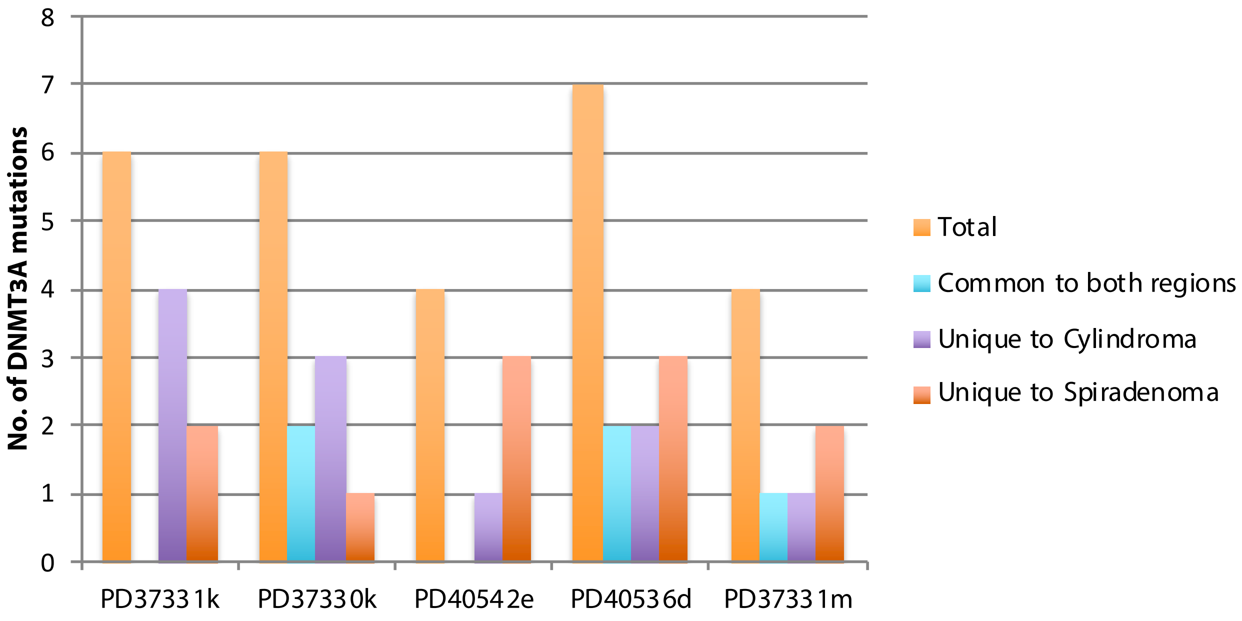

b

Sample	Mutation	Cylindroma VAF	Spiradenoma VAF
PD37331k	p.A70V	5%	0%
	p.G83R	5%	0%
	p.I539V	0%	2%
	p.R635G	0%	4%
	p.I655T	7%	0%
	p.I661fs	11%	0%
PD37330k	p.E61V	0%	5%
	p.S62G	3%	3%
	p.S73P	3%	1%
	p.D92fs	3%	0%
	p.D668V	8%	0%
	pR729Q	43%	26%
PD40542e	p.R23X	4%	0%
	p.V60E	0%	8%
	p.V71M	0%	5%
	p.E205X	0%	3%
PD40536d	p.K74E	2%	6%
	c.1123-2A>C	5%	21%
	p.V622A	0%	3%
	p.I633fs	0%	4%
	p.E667V	8%	0%
	p.G673A	7%	0%
	p.R729W	2%	7%
PD37331m	p.K67E	5%	0%
	p.K299R	1%	2%
	p.K651R	0%	7%
	p.C666R	0%	7%

Supplementary Figure 4. Intratumoral targeted deep sequencing. a DNA extracted from microdissected regions of 5 tumors with cylindroma and spiradenoma histophenotypes in the same section show the presence of the same driver mutation in 3 out of 5 cases, at varying VAFs, consistent with the contiguous growth of these tumors in three-dimensional space. **b** Table of *DNMT3A* mutations.

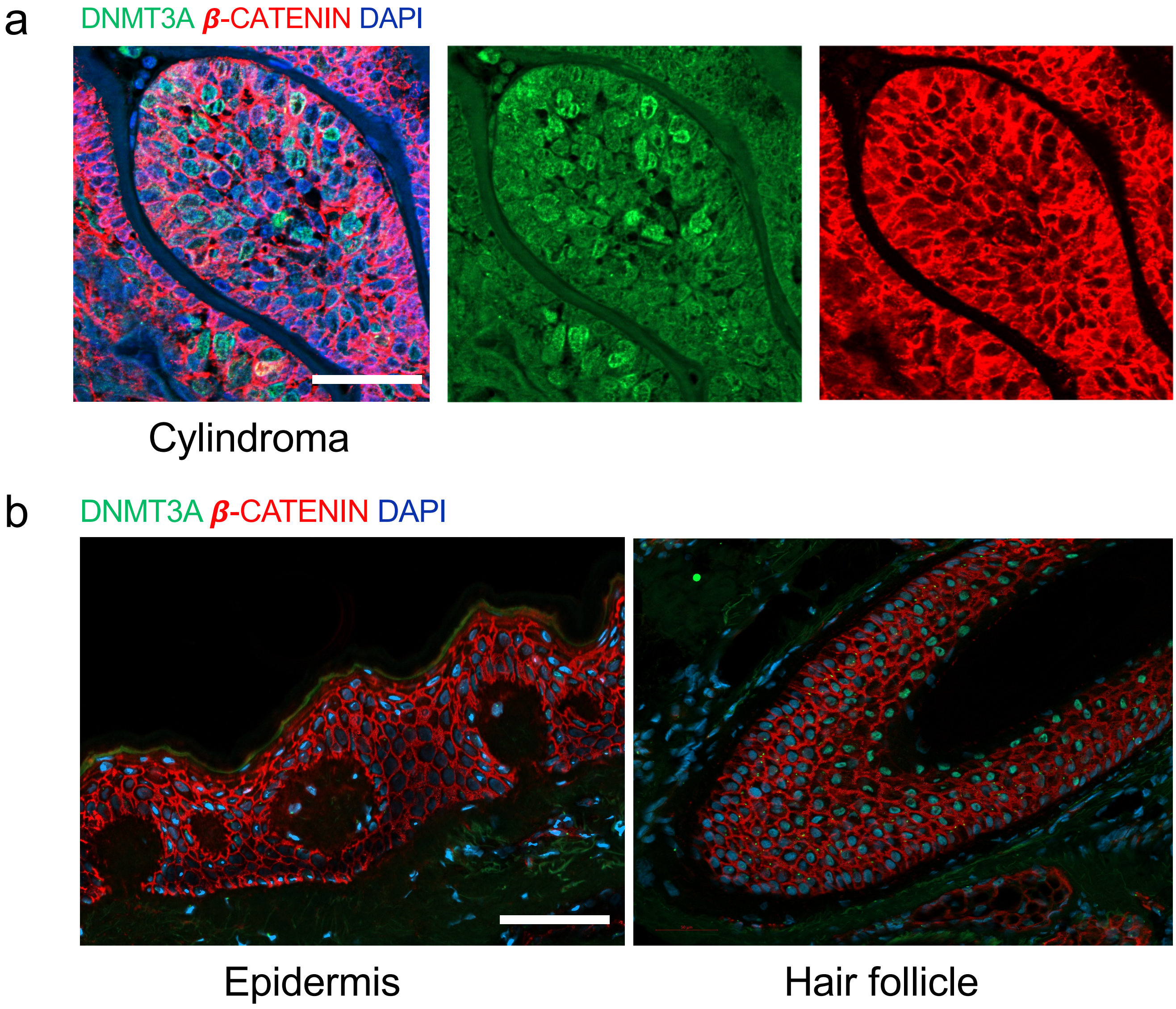

Supplementary Figure 5. DNMT3A expression in CCS tumors. a DNMT3A and beta-catenin expression in a cylindroma tumour. **b** Low DNMT3A expression and membranous beta-catenin expression in control epidermis (left panel) and hair follicles (right panel) (White scale bar=50μm).

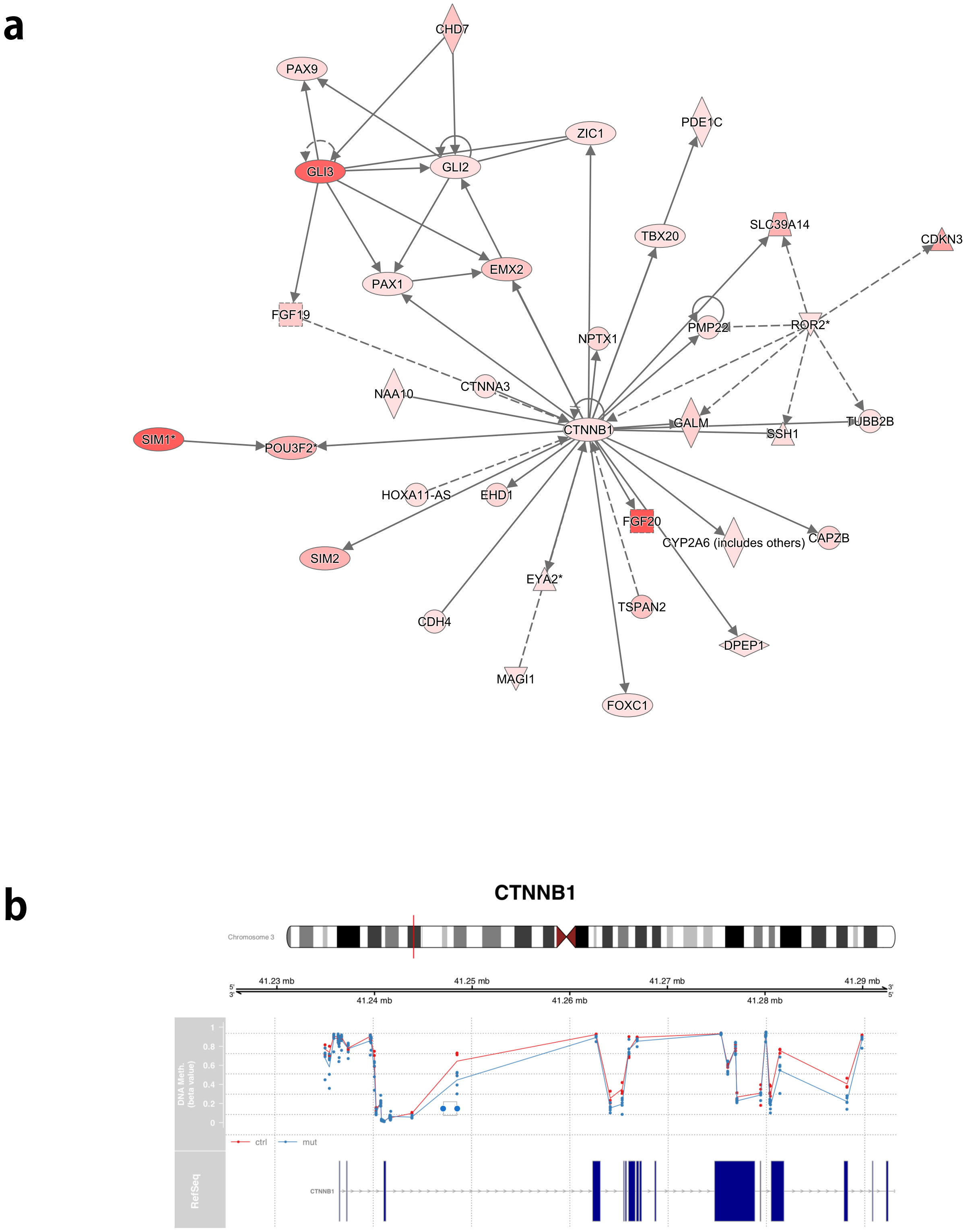

Supplementary Figure 6. ***DNMT3A2*** **mutated tumors are associated with a network of hypomethylated genes with functional relationships to beta-catenin in CCS tumors. a** Pathway analysis of hypomethylated genes in a cluster of 5 tumours carrying *DNMT3A2* mutant VAF of >0.05 highlighted a network of genes functionally related to ß-catenin, indicated in a network diagram. Deeper pink indicates increased hypomethylation. Solid lines indicate direct interactions, and dashed lines indicate indirect interactions. **b** Methylation profile of beta-catenin is indicated at probe-level across 8 tumours.

a

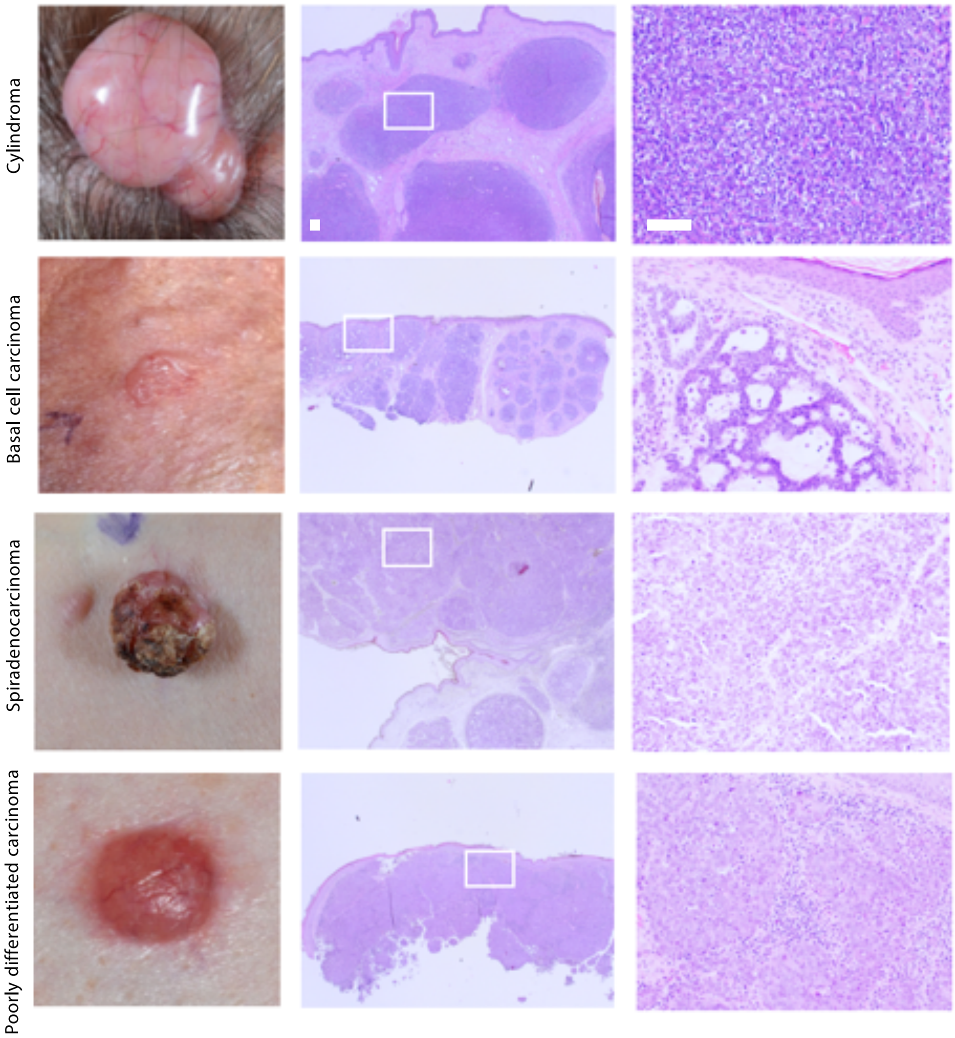

CYLD	*Putative drivers*
LOH	*BCOR*<br>*DNMT3A*
LOH	*PTCH1*
LOH	*MBD4*<br>*CREBBP*<br>*KDM6A*<br>*NOTCH2*<br>*BAP1*
No LOH	*EP300*<br>*TP53*

Supplementary Figure 7. Clinical and histological features of malignant tumors seen in CCS. a Cutaneous tumors seen in CCS, with histological features demonstrated in adjacent panels, with driver mutations summarised. Cylindroma, typically a pink nodule with overlying vessels, demonstrate epithelial cells arranged in cylinders. Malignant spiradenocarcinoma, poorly differentiated adenocarcinoma and basal cell carcinoma demonstrate clinical, histological and genetic differences from this benign tumor (White scale bar=50μm; LOH=Loss of heterozygosity).

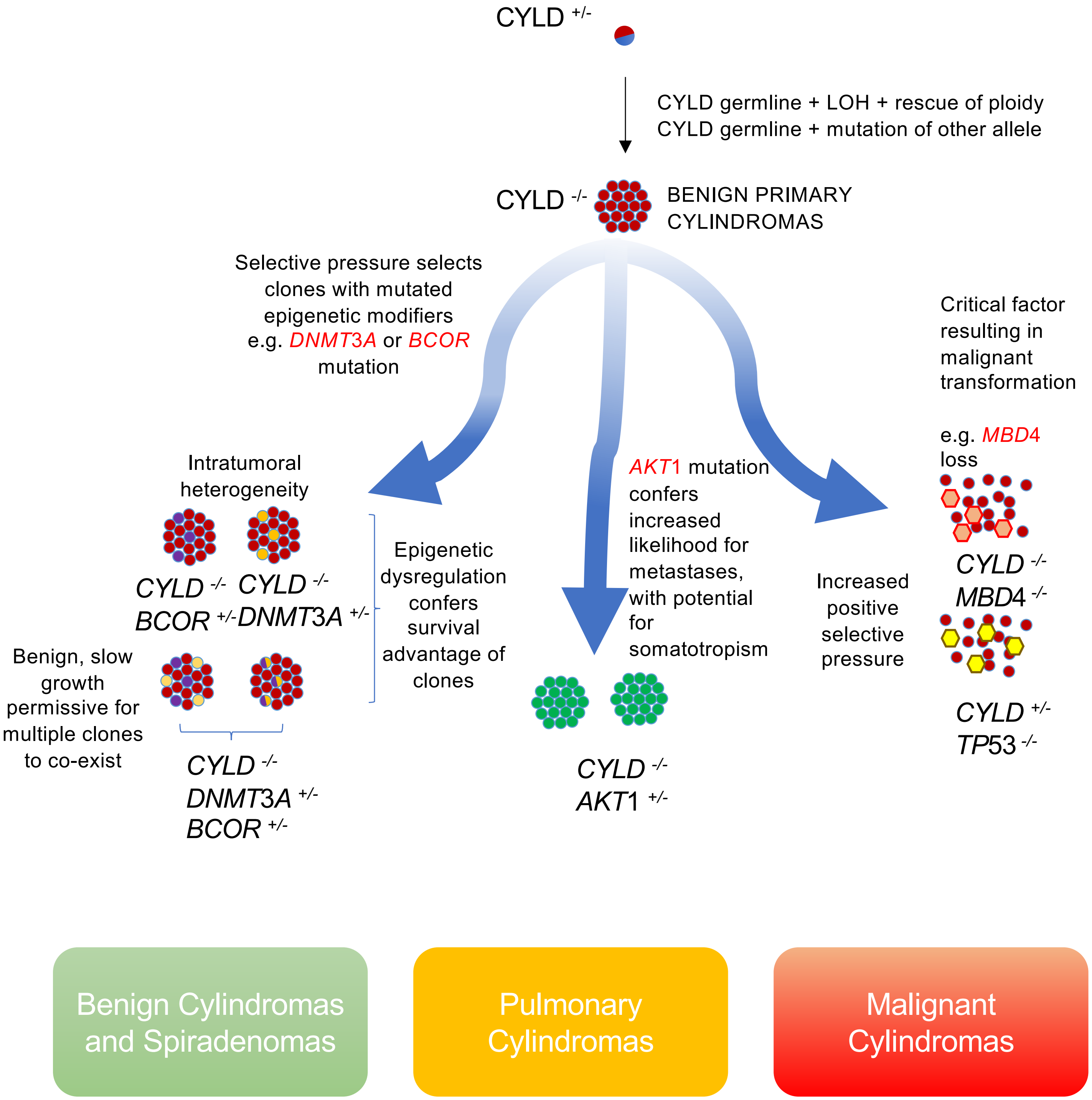

Supplementary Figure 8. A model of mutation acquisition in CCS tumors.

Sample	Gene	CDS	Protein	Type	Effect	VAF
PD37330h	*AKT1*	c.49G>A	p.E17K	Sub	missense	0.22
PD37331f	*AKT1*	c.49G>A	p.E17K	Sub	missense	0.55
PD37331g	*AKT1*	c.49G>A	p.E17K	Sub	missense	0.44
PD37331h	*AKT1*	c.49G>A	p.E17K	Sub	missense	0.31
PD36119a	*BAP1*	c.2036_2055del20	p.I679fs*31	Del	frameshift	0.72
PD37330a	*BCOR*	c.2606_2607insAGA	p.Y869delins*	Ins	nonsense	0.31
PD37330h	*BCOR*	c.1722_1723insC	p.N575fs*36	Ins	frameshift	0.087
PD37330i	*BCOR*	c.1061_1062insAA	p.Y354fs*1	Ins	frameshift	0.378
PD37330j	*BCOR*	c.2221_2225delGAGAAinsTTTC	p.E741fs*45	Complex	frameshift	0.309
PD40537a	*BCOR*	c.1059delC	p.Y354fs*24	Del	frameshift	0.074
PD40540c	*BCOR*	c.3621_3622insA	p.Q1208fs*8	Ins	frameshift	0.075
PD40543a	*BCOR*	c.2428C>T	p.R810*	Sub	nonsense	0.048
PD40545a	*BCOR*	c.1572_1573insTGGCAAAAGC	p.M525fs*35	Ins	frameshift	0.350
PD36119a	*CREBBP*	c.4337G>A	p.R1446H	Sub	missense	0.31
PD36119a	*CREBBP*	c.3307C>T	p.R1103*	Sub	nonsense	0.39
PD37330a	*DNMT3A*	c.1518C>G	p.H506Q	Sub	missense	0.36
PD37330a	*DNMT3A*	c.1517A>G	p.H506R	Sub	missense	0.37
PD37330c	*DNMT3A*	c.1531G>A	p.G511R	Sub	missense	0.42
PD37330g	*DNMT3A*	c.2339T>C	p.I780T	Sub	missense	0.34
PD40536d	*DNMT3A*	c.1123-2A>C	p.?	Sub	ess_splice	0.095
PD40537a	*DNMT3A*	c.2644C>T	p.R882C	Sub	missense	0.26
PD40541a	*DNMT3A*	c.2638delA	p.M880fs*1	Del	frameshift	0.182
PD40536c	*EP300*	c.584C>A	p.S195*	Sub	nonsense	0.085
PD40536c	*EP300*	c.2855C>G	p.S952*	Sub	nonsense	0.082
PD36119a	*KDM6A*	c.1972C>T	p.R658*	Sub	nonsense	0.65
PD36119a	*NOTCH2*	c.6909_6910insC	p.I2304fs*9	Ins	frameshift	0.37
PD40544c	*PTCH1*	c.843_863del21insG	p.L282fs*30	Complex	frameshift	0.320
PD40536c	*TP53*	c.614_615insGTA	p.E204_Y205ins*	Ins	nonsense	0.312

Supplementary Table 1 Variant allele fraction of coding mutations detected using whole genome sequencing and whole exome sequencing

Sample	Source	Mutant reads	Total reads	Variant Allele Fraction
PD37330a	Cylindroma	21	45	0.47
PD37330c	Cylindroma	15	37	0.41
PD37330d	Normal skin	12	31	0.39
PD37330e	Cylindroma	20	35	0.57
PD37330f	Cylindroma	22	50	0.44
PD37330g	Cylindroma	25	45	0.56
PD37331a	Cylindroma	19	44	0.43
PD37331c	Cylindroma	19	38	0.50
PD37331d	Normal skin	18	43	0.42
PD37331e	Normal lung	19	37	0.51
PD37331f	Pulmonary cylindroma	24	43	0.56
PD37331g	Pulmonary cylindroma	22	40	0.55
PD37331h	Pulmonary cylindroma	18	36	0.50
PD37331i	Cylindroma	20	46	0.43
Exome samples				
PD36119a	Malignant cylindroma	166	202	0.82
PD36119c	Cylindroma	81	153	0.53

Supplementary Table 2 *MBD*4 somatic status in tumors from pedigree 1
