# Supplementary material for: Epigenetic modifiers DNMT3A and BCOR are recurrently mutated in CYLD cutaneous syndrome
Source: Nat Commun. 2019 Oct 17;10:4717. doi: 10.1038/s41467-019-12746-w (PMC6797807; doi:10.1038/s41467-019-12746-w)
Supplement: Supplementary file 2 — Description of Additional Supplementary Files [file 41467_2019_12746_MOESM2_ESM.pdf]

Description of Additional Supplementary Files

File Name: Supplementary Data 1

Description: Sample list of studied tumours and patient genotypes

File Name: Supplementary Data 2

Description: A full list of mutations detected in this study

File Name: Supplementary Data 3

Description: A list of of the top 25 networks of related genes identified by Ingenuity Pathway Analysis in the 250 most differentially methylated probes between a cluster of DNMT3A2 mutant tumours and DNMT3A2 wildtype tumours
