# Supplementary material for: Epigenetic modifiers DNMT3A and BCOR are recurrently mutated in CYLD cutaneous syndrome
Source: Nat Commun. 2019 Oct 17;10:4717. doi: 10.1038/s41467-019-12746-w (PMC6797807; doi:10.1038/s41467-019-12746-w)
Supplement: Supplementary file 6 — Reporting Summary [file 41467_2019_12746_MOESM6_ESM.pdf]

nature research

Corresponding author(s): Neil Rajan

Last updated by author(s): Sep 23, 2019

Reporting Summary

Nature Research wishes to improve the reproducibility of the work that we publish. This form provides structure for consistency and transparency in reporting. For further information on Nature Research policies, see Authors & Referees and the Editorial Policy Checklist.

Statistics

For all statistical analyses, confirm that the following items are present in the figure legend, table legend, main text, or Methods section.

n/a	Confirmed	
☐	☒	The exact sample size (*n*) for each experimental group/condition, given as a discrete number and unit of measurement
☒	☐	A statement on whether measurements were taken from distinct samples or whether the same sample was measured repeatedly
☐	☒	The statistical test(s) used AND whether they are one- or two-sided<br>*Only common tests should be described solely by name; describe more complex techniques in the Methods section.*
☒	☐	A description of all covariates tested
☒	☐	A description of any assumptions or corrections, such as tests of normality and adjustment for multiple comparisons
☒	☐	A full description of the statistical parameters including central tendency (e.g. means) or other basic estimates (e.g. regression coefficient) AND variation (e.g. standard deviation) or associated estimates of uncertainty (e.g. confidence intervals)
☒	☐	For null hypothesis testing, the test statistic (e.g. *F*, *t*, *r*) with confidence intervals, effect sizes, degrees of freedom and *P* value noted<br>*Give P values as exact values whenever suitable.*
☒	☐	For Bayesian analysis, information on the choice of priors and Markov chain Monte Carlo settings
☒	☐	For hierarchical and complex designs, identification of the appropriate level for tests and full reporting of outcomes
☒	☐	Estimates of effect sizes (e.g. Cohen's *d*, Pearson's *r*), indicating how they were calculated

Our web collection on statistics for biologists contains articles on many of the points above.

Software and code

Policy information about availability of computer code

Data collection	No software was used
Data analysis	No new code was produced for the analysis of the data in this manuscript. Details of the computer code used are included in the Materials and Methods section.

For manuscripts utilizing custom algorithms or software that are central to the research but not yet described in published literature, software must be made available to editors/reviewers. We strongly encourage code deposition in a community repository (e.g. GitHub). See the Nature Research guidelines for submitting code & software for further information.

Data

Policy information about availability of data

All manuscripts must include a data availability statement. This statement should provide the following information, where applicable:

- Accession codes, unique identifiers, or web links for publicly available datasets
- A list of figures that have associated raw data
- A description of any restrictions on data availability

The whole-genome sequencing data have been deposited in the European Genome-phenome Archive (EGA) database under the accession code EGAD00001004573 [https://ega-archive.org/search-results.php?query=EGAD00001004573]. The whole-exome sequencing data have been deposited in the EGA database under the accession code EGAD00001005305 [https://ega-archive.org/search-results.php?query=EGAD00001005305+]. RNA sequencing data have been deposited in the EGA database under the accession code EGAD00001005305 [https://ega-archive.org/search-results.php?query=EGAD00001005305+]. Targeted deep sequencing data were deposited in the EGA database under the accession code EGAD00001005305 [https://ega-archive.org/search-results.php?query=EGAD00001005305 +Methylation data have been deposited in the A database under the accession code EGAD00010001755 [https://ega-archive.org/search-results.php?query=EGAD00010001755]. All the other data supporting the findings of this study are available within the article and its supplementary information files and from the corresponding author upon reasonable request.

Field-specific reporting

Please select the one below that is the best fit for your research. If you are not sure, read the appropriate sections before making your selection.

☒ Life sciences ☐ Behavioural & social sciences ☐ Ecological, evolutionary & environmental sciences

For a reference copy of the document with all sections, see nature.com/documents/nr-reporting-summary-flat.pdf

Life sciences study design

All studies must disclose on these points even when the disclosure is negative.

Sample size	This study involved 15 genotyped patients with germline CYLD mutations.
Data exclusions	No data was excluded from the analyses
Replication	Replication of mutations in DNMT3A and BCOR found using exome and whole genome assays was performed using a targeted deep sequencing assay.
Randomization	Randomization was not applicable to this study which did not have an intervention, and instead sought to discover driver mutations in CYLD cutaneous syndrome.
Blinding	Blinding was not applicable to this study which did not have an assessment of an intervention , and instead sought to discover driver mutations in CYLD cutaneous syndrome.

Reporting for specific materials, systems and methods

We require information from authors about some types of materials, experimental systems and methods used in many studies. Here, indicate whether each material, system or method listed is relevant to your study. If you are not sure if a list item applies to your research, read the appropriate section before selecting a response.

Materials & experimental systems

n/a	Involved in the study	
☐	☒	Antibodies
☒	☐	Eukaryotic cell lines
☒	☐	Palaeontology
☒	☐	Animals and other organisms
☐	☒	Human research participants
☒	☐	Clinical data

Methods

n/a	Involved in the study	
☒	☐	ChIP-seq
☒	☐	Flow cytometry
☒	☐	MRI-based neuroimaging

Antibodies

Antibodies used	Antibodies against DNMT3A (#3598) and Ki-67 (#9449) were obtained from Cell Signalling, USA. Beta Catenin antibody (#610153) was obtained from BD Transduction USA. Secondary fluorescent antibodies (Alexa Fluor #111-545-144 488-conjugated Goat-Anti Rabbit and #115-585-146 594-conjugated Goat-Anti Mouse)
Validation	Primary antibodies were validated using appropriate positive and negative controls, and were used as per manufacturer's specifications.

Human research participants

Policy information about studies involving human research participants

Population characteristics	This study involved 15 genotyped patients with germline CYLD mutations. Age and gender data are included in Supplementary Data 1.
Recruitment	Retrospective review of the case notes and radiological data of 15 genotyped CYLD mutation carriers that were under follow up between 1 July 2013 and 1 July 2017 was performed. Skin and lung samples were obtained from patients with signed, informed, consent and details of samples are shown in Supplementary Data 1.
Ethics oversight	Research ethics committee approval was obtained from the Hartlepool Research Ethics Committee and North East – Newcastle & North Tyneside 1 Research Ethics Committee for this work (REC Ref: 06/Q1001/59; 08/H0906/95+5).

Note that full information on the approval of the study protocol must also be provided in the manuscript.
